# Crystal structure of 1-(4-methyl­phen­yl)-3-(propan-2-yl­idene­amino)­thio­urea

**DOI:** 10.1107/S2056989015017624

**Published:** 2015-09-26

**Authors:** Mukesh M. Jotani, Chien Ing Yeo, Edward R. T. Tiekink

**Affiliations:** a Department of Physics, Bhavan’s Sheth R. A. College of Science, Ahmedabad, Gujarat 380001, India; b Department of Chemistry, University of Malaya, 50603 Kuala Lumpur, Malaysia; c Centre for Chemical Crystallography, Faculty of Science and Technology, Sunway University, 47500 Bandar Sunway, Selangor Darul Ehsan, Malaysia

**Keywords:** crystal structure, hydrogen bonding, thio­urea derivative, thio­semicarbazone, Hirshfeld surface analysis

## Abstract

In the title thio­semicarbazone, C_11_H_15_N_3_S, the *p*-tolyl-N—H and imino-N—H groups are *anti* and *syn*, respectively, to the central thione-S atom. This allows for the formation of an intra­molecular *p*-tolyl-N—H⋯N(imino) hydrogen bond. The mol­ecule is twisted with the dihedral angle between the *p*-tolyl ring and the non-hydrogen atoms of the N=CMe_2_ residue being 29.27 (8)°. The crystal packing features supra­molecular layers lying in the *bc* plane whereby centrosymmetric aggregates sustained by eight-membered thio­amide {⋯HNCS}_2_ synthons are linked by further N—H⋯S hydrogen bonds. Layers are connected *via* methyl-C—H⋯π inter­actions. The supra­molecular aggregation was further investigated by an analysis of the Hirshfeld surface and comparison made to related structures.

## Abstract 2

The title twisted thio­semicarbazone mol­ecule has, respectively, *anti-* and *syn*-dispositions of the *p*-tolyl-N—H and imino-N—H groups with respect to the central thione-S atom allowing for the formation of an intra­molecular *p*-tolyl-N—H⋯N(imino) hydrogen bond. The presence of N—H⋯S hydrogen bonds lead to layers in the *bc* plane which are connected by methyl-C—H⋯π inter­actions.

## Chemical context   

The reaction between an alcohol or amine (primary or secondary) with *N*-alkyl- or *N*-aryl-iso­thio­cyanides usually results in the formation of thio­carbamides. For example, in the case of reactions involving a monofunctional alcohol, the reaction proceeds in the following manner: *R*—OH + *R*′N=C=S → *R*OC(=S)N(H)*R*′ (Ho *et al.*, 2005 ▸). Often, reactions are facilitated by initially employing an alkali metal hydroxide as the base and later adding an acid, *e.g*. HCl (Ho *et al.*, 2005 ▸). Such mol­ecules are of inter­est as when deprotonated, they can function as effective thiol­ate ligands for phosphanegold(I) derivatives, which display biological activity. For example, Ph_3_PAu[SC(O–alk­yl)=N(ar­yl)] com­pounds exhibit significant cytotoxicity against a variety of cancer cell lines and mechanistic studies show that they can kill cancer cells by initiating a variety of apoptotic pathways, both extrinsic and intrinsic (Yeo, Ooi *et al.*, 2013 ▸; Ooi, Yeo *et al.*, 2015 ▸). Related species, *i.e*. Ph_3_PAu[SC(O–alk­yl)=N(*p*-tol­yl)], exhibit potency against Gram-positive bacteria (Yeo, Sim *et al.*, 2013 ▸). Over and above these considerations, systematic studies into the structural chemistry of these mol­ecules, which have proven relatively easy to crystallize, have been of some inter­est in crystal engineering endeavours (Ho *et al.*, 2006 ▸; Kuan *et al.*, 2008 ▸). In the course of studies to increase the functionality of the thio­carbamide mol­ecules, bipodal {1,4-[MeOC(=S)N(H)]_2_C_6_H_4_} was successfully synthesized along with binuclear phosphanegold(I) complexes (Yeo, Khoo *et al.*, 2015 ▸). Recent attempts at expanding this chemistry by using thio­urea as an amine donor have been undertaken. As reported very recently, 1:2 reactions between thio­urea and *R*′N=C=S resulted in the isolation of salts containing 1,2,3-thia­zole-based cations resulting from 1:1 cyclizations (Yeo, Tan *et al.*, 2015 ▸). Herein, the product of an analogous reaction involving a bifunctional amine, *i.e*. H_2_NNH_2_ (hydrazine) with (*p*-tol­yl)N=C=S, conducted in acetone solution, is described, namely the thio­semicarbazone, (I). Mol­ecules related to (I) and especially their metal complexes continue to attract attention owing to potential biological activity (Dilworth & Hueting, 2012 ▸; Lukmantara *et al.*, 2013 ▸; Su *et al.*, 2013 ▸).
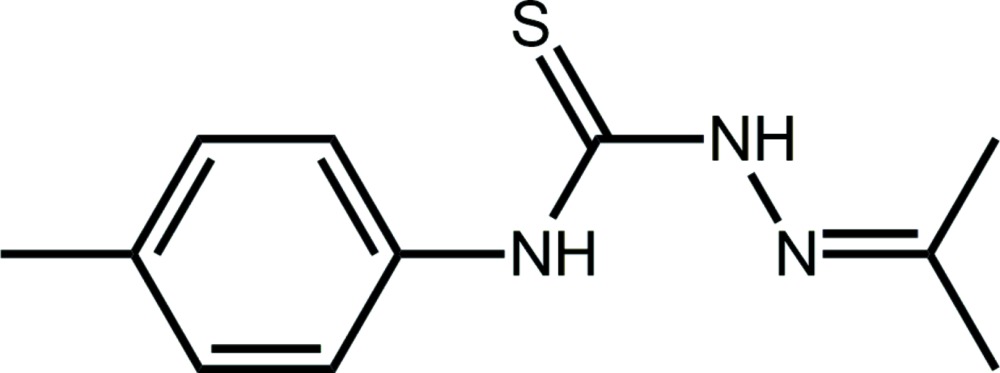


### Structural commentary   

**Figure 1:**
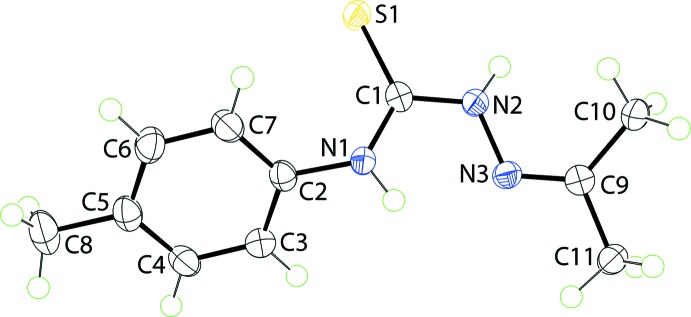
The mol­ecular structure of (I) showing displacement ellipsoids at the 70% probability level.

The mol­ecular structure of (I), Fig. 1 ▸, comprises three planar regions. The central NC(=S)N chromophore (the r.m.s. deviation of the fitted atoms is 0.0020 Å) has *anti*- and *syn*-dispositions of the N1- and N2-bound H atoms, respectively, with respect to the central thione-S1 atom. The N1-bound H atom is *syn* to the imino-N3 atom allowing for the formation of a five-membered loop *via* an N1—H⋯N3 hydrogen bond, Table 1 ▸. The central plane forms dihedral angles of 23.49 (4)° with the propan-2-yl­idene­amino residue (N=CMe_2_; r.m.s. deviation for the C_3_N atoms = 0.0002 Å) and 43.30 (5)° with the *p*-tolyl ring. Overall, the mol­ecule is twisted as qu­anti­fied by the dihedral angle between the outer planes of 29.27 (8)°.

**Figure 2:**
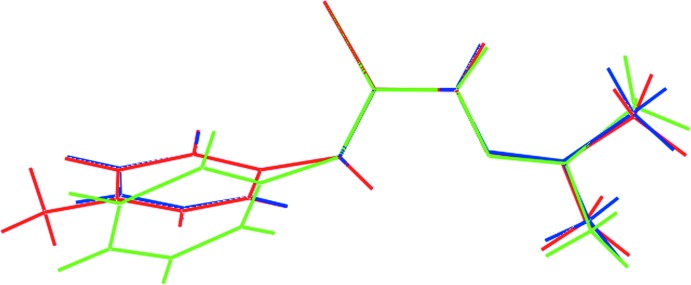
Overlay diagram of the mol­ecules in (I), red image, and (II), forms *a* (green) and *b* (blue). The mol­ecules have been overlapped so that the central NC(=S)N chromophores are coincident.

Two *P*2_1_/*c* polymorphs have been reported for the parent compound, *i.e*. having a phenyl rather than a *p*-tolyl substit­uent (Jian *et al.*, 2005 ▸; Venkatraman *et al.*, 2005 ▸); the structure of (I) also crystallizes in the *P*2_1_/*c* space group. As revealed from the data collated in Table 2 ▸, there is a high degree of concordance in the key bond lengths and angles for the three mol­ecules, as might be expected. However, there are some notable differences in the torsion-angle data as well as in the dihedral angles between the three least-squares planes discussed above, Table 2 ▸. From these and the overlay diagram shown in Fig. 2 ▸, it is apparent that the mol­ecular structure of (I) more closely matches that observed in the polymorph reported by Venkatraman *et al.* (2005 ▸) rather than that described by Jian *et al.* (2005 ▸). This conclusion is also vindicated in the unit cell data, *i.e. a* = 12.225 (3), *b* = 7.618 (2), *c* = 11.639 (3) Å, β = 102.660 (4)° reported for the former (Venkatraman *et al.*, 2005 ▸).

## NMR invesitgations   

The conformation of (I) was also investigated in CDCl_3_ solution by NMR methods. Assignments were made with the aid of the inter­pretative program, *Chemdraw Ultra* (CambridgeSoft Corporation, 2002 ▸). On the basis of multiple ^1^H and ^13^C{^1^H} resonances for the methyl groups of the propan-2-yl­idene­amino residue, it appears that the (propan-2-yl­idene­amino)­thio­urea residue has a locked configuration, consistent with the persistence of the intra­molecular N1—H⋯N3 hydrogen bond in CDCl_3_ solution.

## Supra­molecular features   

**Figure 3:**
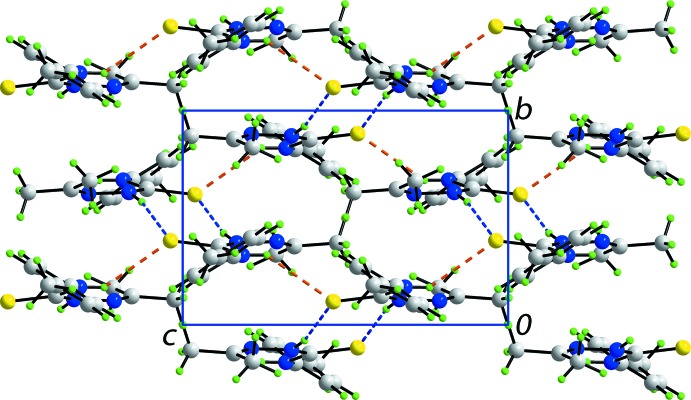
Supra­molecular layer in the *bc* plane in the crystal packing of (I). Centrosymmetric aggregates mediated by eight-membered thio­amide {⋯HNCS}_2_ synthons (shown as orange dashed lines) are linked by additional amide-N—H⋯S hydrogen bonds, shown as blue dashed lines.

**Figure 4:**
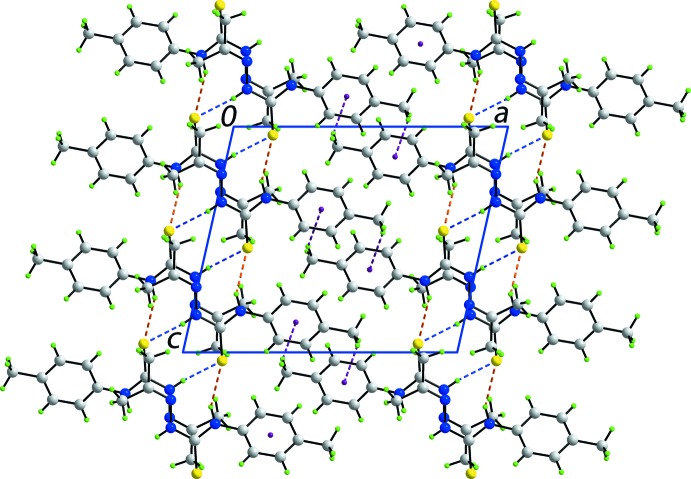
A view of the unit cell contents of (I) shown in projection down the *b* axis. Supra­molecular layers, illustrated in Fig. 3 ▸, are linked *via* C—H⋯π inter­actions, shown as purple dashed lines, leading to a three-dimensional architecture.

In the crystal, N—H⋯S and C—H⋯π inter­actions provide identifiable points of contact between mol­ecules; these inter­actions are qu­anti­fied in Table 1 ▸. Centrosymmetrically related mol­ecules are connected by pairs of amide-N2—H⋯S1 hydrogen bonds, forming eight-membered thio­amide {⋯HNCS}_2_ synthons. These are connected into supra­molecular layers in the *bc* plane by amide-N1—H⋯S1 hydrogen bonds so that the S1 atom accepts two hydrogen bonds, Fig. 3 ▸. The *p*-tolyl groups protrude to either side of each layer and inter-digitate along the *a* axis with adjacent layers allowing for the formation of methyl-C8—H⋯π(C2–C7) inter­actions, thereby consolidating the three-dimensional architecture, Fig. 4 ▸.

## Analysis of the Hirshfeld surfaces   

The crystal packing was further investigated by an analysis of the Hirshfeld surface (Spackman & Jayatilaka, 2009 ▸) employing *CrystalExplorer* (Wolff *et al.*, 2012 ▸). Fingerprint plots (Rohl *et al.*, 2008 ▸) were calculated, as were the electrostatic potentials using *TONTO* (Spackman *et al.*, 2008 ▸; Jayatilaka *et al.*, 2005 ▸) integrated into *CrystalExplorer*; the electrostatic potentials were mapped on the Hirshfeld surfaces using the STO–3G basis set at the level of Hartree–Fock theory over a range of ±0.075 au.

**Figure 5:**
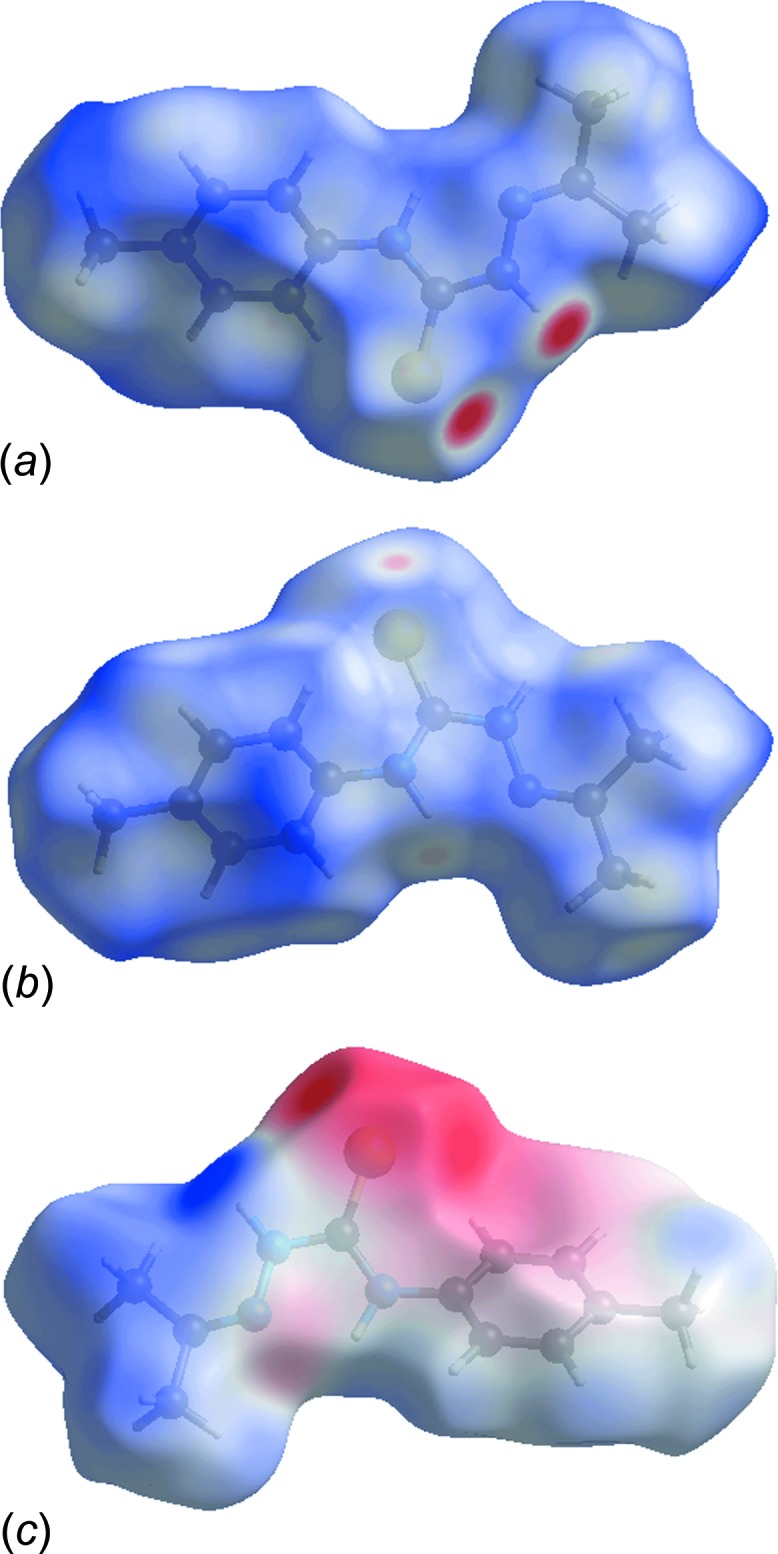
Views of the Hirshfeld surface for (I): (*a*) and (*b*) mapped over *d*_norm_, and (*c*) mapped over the calculated electrostatic potential.

Two views of the Hirshfeld surface mapped over *d*_norm_ are shown in Fig. 5 ▸ *a* and *b*. The deep-red depressions at the S1 and N2 atoms (Fig. 5 ▸ *a*) confirm their role as an acceptor and donor in the hydrogen-bonding scheme, respectively. This is also evident from the dark-red and blue regions, respectively, on the Hirshfeld surface mapped over the calculated electrostatic potential (Fig. 5 ▸ *c*). The diminutive red spots near S1 and N1 (Fig. 5 ▸ *b*) indicate their involvement in the inter­molecular N—H⋯S hydrogen bond.

**Figure 6:**
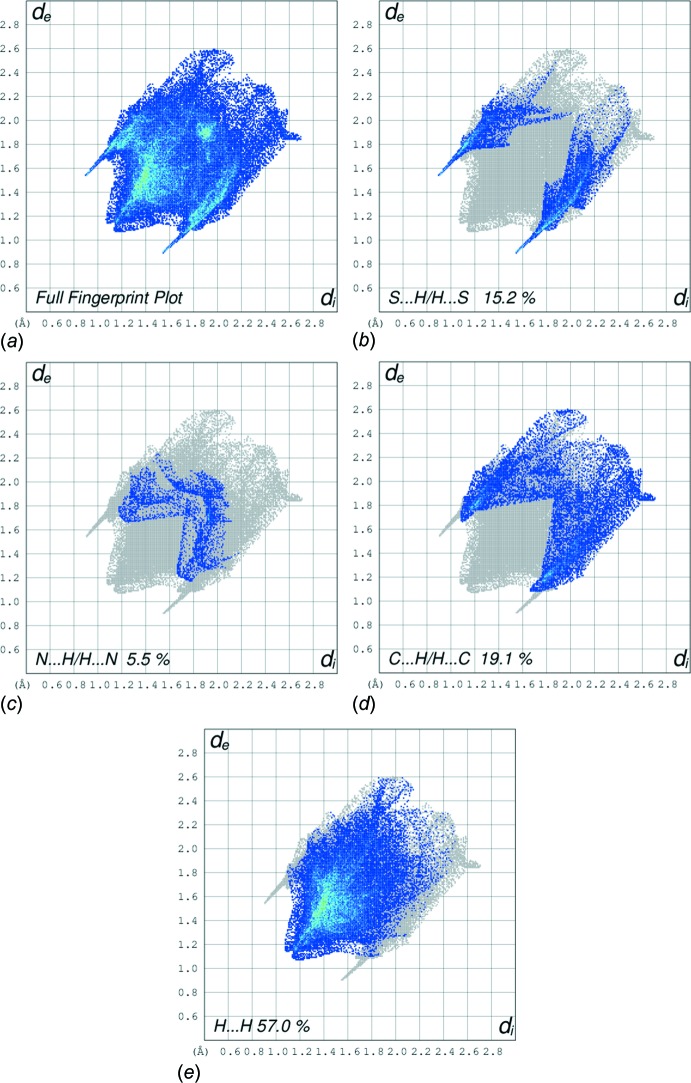
2D Fingerprint plots for (I): (*a*) full, (*b*) delineated to show S⋯H/H⋯S, (*c*) N⋯H/H⋯N, (*d*) C⋯H/H⋯C, and (*e*) H⋯H inter­actions.

**Figure 7:**
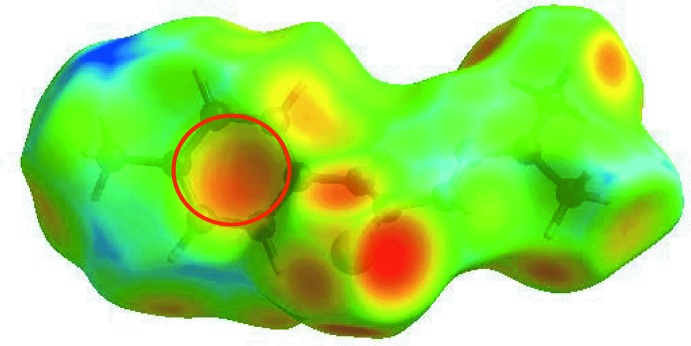
View of the Hirshfeld surface for (I) mapped over *d*_e_.

**Figure 8:**
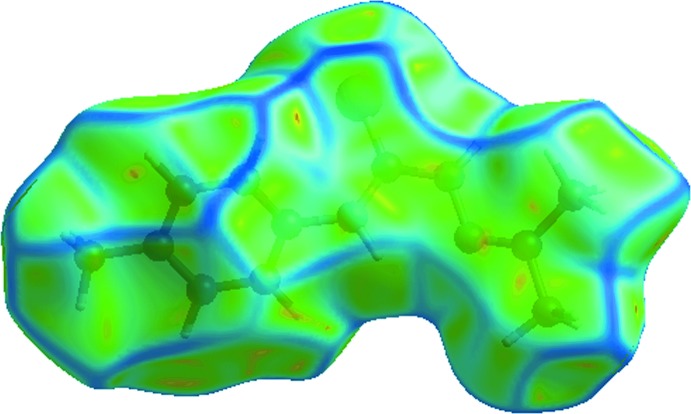
Hirshfeld surfaces for (I) mapped over curvedness.

The overall two-dimensional fingerprint plot (Fig. 6 ▸ *a*) and those delineated into H⋯H, S⋯H/H⋯S, N⋯H/H⋯N and C⋯H/H⋯C H⋯H (Fig. 6 ▸ *b*–*d*, respectively) inter­actions operating in the crystal structure of (I) are illustrated in Fig. 6 ▸; the relative contributions are summarized in Table 3 ▸. The prominent pair of sharp spikes of equal length (*d*_e_ + *d*_i_ = 2.45 Å; Fig. 6 ▸ *b*) with a 15.2% contribution due to S⋯H/H⋯S contacts to the Hirshfeld surfaces also suggest the presence of these inter­actions in the crystal packing. The light-red region near N3 (Fig. 5 ▸ *a*) and diminutive red spot near N1—H (Fig. 5 ▸ *b*) are consistent with relatively smaller contributions from N⋯H/H⋯N contacts, *i.e*. 2.5 and 3.0%, respectively, and are indicative of the weak intra­molecular hydrogen bond. The strength of such an inter­action can be visualized from the 2D fingerprint plot corresponding to N⋯H/ H⋯N contacts (Fig. 6 ▸ *c*). The bright-orange spot in the surface mapped with *d*_e_ (within a red circle in Fig. 7 ▸) about the aryl ring and a light-blue region around the tolyl-hydrogen atom, H8*C* (Fig. 7 ▸), suggest a contribution from the C—H⋯π inter­action (Table 1 ▸). This is also evident through distinct pair of ‘wings’ in the fingerprint plot corresponding to C⋯H/H⋯C contacts (Fig. 6 ▸ *d*). The wing at the top, left belongs to C—H donors, while that at the bottom, right corresponds to the surface around π-acceptors with 11.3 and 7.8% contribution from C⋯H and H⋯C contacts, respectively. The H⋯H contacts reflected in the middle of scattered points in Fig. 6 ▸ *e* provide the most significant contribution, *i.e*. 57.0%, to the Hirshfeld surface arising from a side-ways approach. The small, flat segments delineated by the blue outline in the surface mapped with curvedness (Fig. 8 ▸) and the small (*i.e*. 0.7%) contribution from C⋯C contacts to the surface indicates the absence of π–π stacking inter­actions in the structure.

## Database survey   

According to a search of the Cambridge Structural Database (Groom & Allen, 2014 ▸), there are no direct analogues of (I), either in the coordinated or uncoordinated form. As mentioned in the *Structural commentary*, the parent compound has been characterized in two polymorphic forms (Jian *et al.*, 2005 ▸; Venkatraman *et al.*, 2005 ▸). The parent compound, LH, has also been observed to coordinate metal centres. Thus, monodentate coordination *via* the thione-S atom was observed in a neutral complex [ZnCl_2_(LH)_2_] (Bel’skii *et al.*, 1987 ▸). By contrast, a chelating mode *via* thione-S and imino-N atoms was observed in each of the charged complexes [CoBr(LH)_2_]Br (Dessy *et al.*, 1978 ▸) and [(η^6^-*p*-cymene)RuCl(LH)]Cl (Su *et al.*, 2013 ▸). The most closely related structure having the *p*-tolyl substituent but variations at the imino-N atom is one where one methyl group has been substituted by a phenyl (Zhang *et al.*, 2011 ▸). This is also a twisted mol­ecule with the dihedral angle between the *p*-tolyl and NC_3_ residues being 65.44 (7)°.

## Synthesis and crystallization   

To *p*-tolyl iso­thio­cyanate (Sigma–Aldrich; 10 mmol, 1.49 g) in acetone (20 ml) was added hydrazine monohydrate (Sigma–Aldrich; 10 mmol, 0.76 ml). The resulting mixture was stirred for 4 h at room temperature. Both chloro­form (20 ml) and aceto­nitrile (20 ml) were then added, and the resulting mixture left for slow evaporation. Light-brown crystals were obtained after 2 weeks. Yield: 2.012 g (91%). M.p. 412–413 K. ^1^H NMR (400 MHz, CDCl_3_, 298 K): 9.19 (*s*, *br*, 1H, NH—N), 8.56 (*s*, *br*, 1H, NH), 7.49 (*d*, 2H, *m*-aryl, *J* = 8.28 Hz), 7.17 (*d*, 2H, *o*-aryl, *J* = 8.24 Hz), 2.34 (*s*, 3H, aryl-CH_3_), 2.05 (*s*, 3H, CH_3_), 1.94 (*s*, 3H, CH_3_). ^13^C NMR (400 MHz, CDCl_3_, 298 K): 176.4 [CS], 149.6 [C(CH_3_)_2_], 135.8 [C_*ipso*_], 135.4 [C_*para*_], 129.3 [C_*meta*_], 124.5 [C_*ortho*_], 25.3 [CH_3_], 21.0 [aryl-CH_3_], 16.9 [CH_3_, *syn* to N—H]. IR (cm^−1^): ν(N—H) 3240, 3168 (*m*), ν(C=N) 1514 (*vs*), ν(C—N) 1267 (*s*), ν(C=S) 1188 (*vs*).

## Refinement   

Crystal data, data collection and structure refinement details are summarized in Table 4 ▸. Carbon-bound H-atoms were placed in calculated positions (C—H = 0.95–0.98 Å) and were included in the refinement in the riding model approximation, with *U*_iso_(H) set to 1.2–1.5*U*_eq_(C). The N-bound H atoms were located in a difference Fourier map but were refined with a distance restraint of N—H = 0.88±0.01 Å, and with *U*_iso_(H) set to 1.2*U*_eq_(N).

## Supplementary Material

Crystal structure: contains datablock(s) I, global. DOI: 10.1107/S2056989015017624/hb7507sup1.cif

Structure factors: contains datablock(s) I. DOI: 10.1107/S2056989015017624/hb7507Isup2.hkl

Supporting information file. DOI: 10.1107/S2056989015017624/hb7507Isup3.cml

CCDC reference: 1425975

Additional supporting information:  crystallographic information; 3D view; checkCIF report

## Figures and Tables

**Table 1:** Hydrogen-bond geometry (, ) *Cg*1 is the centroid of the C2C7 ring.

*D*H*A*	*D*H	H*A*	*D* *A*	*D*H*A*
N1H1*N*N3	0.88(1)	2.09(2)	2.572(2)	114(1)
N1H1*N*S1^i^	0.88(1)	2.87(2)	3.5618(17)	137(2)
N2H2*N*S1^ii^	0.88(2)	2.57(2)	3.4373(16)	169(2)
C8H8*C* *Cg*1^iii^	0.98	2.81	3.716(2)	154

Symmetry codes: (i) 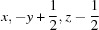; (ii) 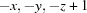; (iii) 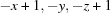.

**Table 2:** Geometric data (, ) for (I) and two polymorphs of (II)

Parameter	(I)	Form *a* of (II)*^*a*^*	Form *b* of (II)*^*b*^*
N2N3	1.397(2)	1.386(2)	1.392(2)
C1S1	1.6873(18)	1.6816(17)	1.6826(17)
C1N1	1.350(2)	1.337(2)	1.343(2)
C1N2	1.350(2)	1.359(2)	1.348(2)
C2N1	1.422(2)	1.420(2)	1.425(2)
C9N3	1.280(2)	1.279(2)	1.276(2)
			
C1N1C2	127.79(16)	129.98(14)	127.97(14)
C1N2N3	117.72(15)	118.17(14)	118.33(14)
N2N3C9	117.91(15)	118.85(15)	117.73(14)
S1C1N1	125.45(14)	126.00(13)	125.75(13)
S1C1N2	120.00(14)	119.37(13)	119.50(12)
N1C1N2	114.54(16)	114.63(15)	114.74(15)
			
S1C1N2N3	169.57(12)	177.46(12)	170.56(12)
C1N1C2C3	132.2(2)	153.87(18)	131.10(19)
C1N2N3C9	165.78(16)	168.43(16)	165.85(15)
			
CN_2_S / C_3_N	23.49(4)	13.19(8)	22.42(9)
CN_2_S / aryl	43.30(5)	39.26(6)	43.90(6)
C_3_N / aryl	29.27(8)	40.15(8)	30.18(8)

Notes: (*a*) Jian *et al.* (2005 ▸); (*b*) Venkatraman *et al.* (2005 ▸).

**Table 3:** Relative contribution (%) to intermolecular interactions calculated from the Hirshfeld surface

Contact	Contribution
HH	57.0
SH/HS	15.2
NH/HN	5.5
CH/HC	19.1
CC	0.7
NN	1.4
CN	0.8
CS	0.2
others	0.1

**Table 4:** Experimental details

Crystal data	
Chemical formula	C_11_H_15_N_3_S
*M*_r_	221.32
Crystal system, space group	Monoclinic, *P*2_1_/*c*
Temperature (K)	100
*a*, *b*, *c* ()	13.7289(13), 7.4341(7), 11.5757(11)
()	102.690(1)
*V* (^3^)	1152.58(19)
*Z*	4
Radiation type	Mo *K*
(mm^1^)	0.25
Crystal size (mm)	0.12 0.05 0.03

Data collection	
Diffractometer	Bruker SMART APEX CCD
Absorption correction	Multi-scan (*SADABS*; Sheldrick, 1996 ▸)
*T*_min_, *T*_max_	0.970, 0.993
No. of measured, independent and observed [*I* > 2(*I*)] reflections	10739, 2646, 2052
*R*_int_	0.050
(sin /)_max_ (^1^)	0.650

Refinement	
*R*[*F* ^2^ > 2(*F* ^2^)], *wR*(*F* ^2^), *S*	0.041, 0.098, 1.02
No. of reflections	2646
No. of parameters	147
No. of restraints	2
H-atom treatment	H atoms treated by a mixture of independent and constrained refinement
_max_, _min_ (e ^3^)	0.27, 0.27

Computer programs: *APEX2* and *SAINT* (Bruker, 2008 ▸), *SHELXS97* (Sheldrick, 2008 ▸), *SHELXL2014*/7 (Sheldrick, 2015 ▸), *ORTEP-3 for Windows* (Farrugia, 2012 ▸), *QMol* (Gans Shalloway, 2001 ▸), *DIAMOND* (Brandenburg, 2006 ▸) and *publCIF* (Westrip, 2010 ▸).

## supplementary crystallographic information

### Crystal data

**Table:**

C_11_H_15_N_3_S	*F*(000) = 472
*M**_r_* = 221.32	*D*_x_ = 1.275 Mg m^−^^3^
Monoclinic, *P*2_1_/*c*	Mo *K*α radiation, λ = 0.71073 Å
*a* = 13.7289 (13) Å	Cell parameters from 1590 reflections
*b* = 7.4341 (7) Å	θ = 3.0–25.5°
*c* = 11.5757 (11) Å	µ = 0.25 mm^−^^1^
β = 102.690 (1)°	*T* = 100 K
*V* = 1152.58 (19) Å^3^	Prism, light-brown
*Z* = 4	0.12 × 0.05 × 0.03 mm

### Data collection

**Table:**

Bruker SMART APEX CCD diffractometer	2646 independent reflections
Radiation source: fine-focus sealed tube	2052 reflections with *I* > 2σ(*I*)
Graphite monochromator	*R*_int_ = 0.050
φ and ω scans	θ_max_ = 27.5°, θ_min_ = 3.0°
Absorption correction: multi-scan (*SADABS*; Sheldrick, 1996)	*h* = −17→17
*T*_min_ = 0.970, *T*_max_ = 0.993	*k* = −9→9
10739 measured reflections	*l* = −13→15

### Refinement

**Table:**

Refinement on *F*^2^	2 restraints
Least-squares matrix: full	Hydrogen site location: mixed
*R*[*F*^2^ > 2σ(*F*^2^)] = 0.041	H atoms treated by a mixture of independent and constrained refinement
*wR*(*F*^2^) = 0.098	*w* = 1/[σ^2^(*F*_o_^2^) + (0.0332*P*)^2^ + 0.7356*P*] where *P* = (*F*_o_^2^ + 2*F*_c_^2^)/3
*S* = 1.02	(Δ/σ)_max_ < 0.001
2646 reflections	Δρ_max_ = 0.27 e Å^−^^3^
147 parameters	Δρ_min_ = −0.27 e Å^−^^3^

### Special details

**Table:**

Geometry. All esds (except the esd in the dihedral angle between two l.s. planes) are estimated using the full covariance matrix. The cell esds are taken into account individually in the estimation of esds in distances, angles and torsion angles; correlations between esds in cell parameters are only used when they are defined by crystal symmetry. An approximate (isotropic) treatment of cell esds is used for estimating esds involving l.s. planes.

### Fractional atomic coordinates and isotropic or equivalent isotropic displacement parameters (Å^2^)

**Table:**

	*x*	*y*	*z*	*U*_iso_*/*U*_eq_	
S1	0.14991 (3)	0.10760 (6)	0.53754 (4)	0.01865 (13)	
N1	0.17657 (11)	0.1754 (2)	0.31707 (14)	0.0179 (3)	
H1N	0.1468 (14)	0.175 (3)	0.2419 (9)	0.023 (6)*	
N2	0.01790 (11)	0.1178 (2)	0.33437 (14)	0.0174 (3)	
H2N	−0.0236 (13)	0.073 (3)	0.3744 (17)	0.032 (6)*	
N3	−0.00516 (11)	0.1118 (2)	0.21071 (13)	0.0177 (3)	
C1	0.11491 (13)	0.1348 (2)	0.38949 (16)	0.0158 (4)	
C2	0.28274 (13)	0.1793 (2)	0.34532 (16)	0.0174 (4)	
C3	0.33249 (14)	0.0928 (3)	0.26883 (17)	0.0218 (4)	
H3	0.2955	0.0313	0.2013	0.026*	
C4	0.43587 (14)	0.0956 (3)	0.29054 (17)	0.0240 (4)	
H4	0.4688	0.0357	0.2374	0.029*	
C5	0.49234 (14)	0.1841 (3)	0.38822 (17)	0.0218 (4)	
C6	0.44118 (15)	0.2713 (3)	0.46362 (18)	0.0240 (4)	
H6	0.4781	0.3329	0.5311	0.029*	
C7	0.33772 (14)	0.2703 (3)	0.44266 (17)	0.0221 (4)	
H7	0.3046	0.3319	0.4949	0.027*	
C8	0.60488 (15)	0.1844 (3)	0.4134 (2)	0.0316 (5)	
H8A	0.6299	0.3006	0.4475	0.047*	
H8B	0.6271	0.1650	0.3395	0.047*	
H8C	0.6307	0.0880	0.4695	0.047*	
C9	−0.09645 (13)	0.1333 (2)	0.15732 (16)	0.0163 (4)	
C10	−0.18214 (14)	0.1717 (3)	0.21420 (17)	0.0220 (4)	
H10A	−0.1571	0.2260	0.2922	0.033*	
H10B	−0.2169	0.0592	0.2233	0.033*	
H10C	−0.2285	0.2549	0.1643	0.033*	
C11	−0.11855 (14)	0.1185 (3)	0.02523 (16)	0.0201 (4)	
H11A	−0.0561	0.1262	−0.0024	0.030*	
H11B	−0.1631	0.2166	−0.0096	0.030*	
H11C	−0.1509	0.0028	0.0011	0.030*	

### Atomic displacement parameters (Å^2^)

**Table:**

	*U*^11^	*U*^22^	*U*^33^	*U*^12^	*U*^13^	*U*^23^
S1	0.0168 (2)	0.0218 (2)	0.0167 (2)	−0.00133 (19)	0.00219 (18)	0.00207 (18)
N1	0.0133 (8)	0.0252 (8)	0.0149 (8)	−0.0017 (6)	0.0020 (6)	0.0013 (7)
N2	0.0148 (8)	0.0226 (8)	0.0149 (8)	−0.0018 (6)	0.0033 (6)	0.0014 (6)
N3	0.0182 (8)	0.0196 (8)	0.0152 (8)	−0.0022 (6)	0.0037 (6)	0.0000 (6)
C1	0.0149 (9)	0.0129 (8)	0.0196 (9)	0.0003 (7)	0.0041 (7)	0.0007 (7)
C2	0.0151 (9)	0.0177 (8)	0.0194 (9)	−0.0003 (7)	0.0040 (7)	0.0046 (7)
C3	0.0200 (10)	0.0272 (10)	0.0181 (10)	−0.0009 (8)	0.0043 (8)	0.0008 (8)
C4	0.0194 (10)	0.0308 (11)	0.0239 (11)	0.0016 (8)	0.0092 (8)	0.0014 (8)
C5	0.0171 (10)	0.0233 (9)	0.0247 (10)	−0.0006 (8)	0.0038 (8)	0.0080 (8)
C6	0.0203 (10)	0.0258 (10)	0.0245 (11)	−0.0051 (8)	0.0018 (8)	−0.0010 (8)
C7	0.0206 (10)	0.0224 (9)	0.0251 (11)	−0.0026 (8)	0.0087 (8)	−0.0040 (8)
C8	0.0172 (10)	0.0364 (12)	0.0408 (13)	−0.0008 (9)	0.0053 (9)	0.0085 (10)
C9	0.0182 (9)	0.0121 (8)	0.0186 (9)	−0.0004 (7)	0.0041 (7)	0.0009 (7)
C10	0.0178 (10)	0.0288 (10)	0.0179 (10)	0.0056 (8)	0.0009 (8)	0.0009 (8)
C11	0.0199 (10)	0.0228 (9)	0.0167 (9)	−0.0008 (8)	0.0020 (8)	0.0004 (7)

### Geometric parameters (Å, º)

**Table:**

S1—C1	1.6873 (18)	C5—C8	1.508 (3)
N1—C1	1.350 (2)	C6—C7	1.387 (3)
N1—C2	1.422 (2)	C6—H6	0.9500
N1—H1N	0.876 (9)	C7—H7	0.9500
N2—C1	1.350 (2)	C8—H8A	0.9800
N2—N3	1.397 (2)	C8—H8B	0.9800
N2—H2N	0.875 (9)	C8—H8C	0.9800
N3—C9	1.280 (2)	C9—C11	1.496 (2)
C2—C7	1.387 (3)	C9—C10	1.496 (3)
C2—C3	1.389 (3)	C10—H10A	0.9800
C3—C4	1.386 (3)	C10—H10B	0.9800
C3—H3	0.9500	C10—H10C	0.9800
C4—C5	1.388 (3)	C11—H11A	0.9800
C4—H4	0.9500	C11—H11B	0.9800
C5—C6	1.395 (3)	C11—H11C	0.9800
			
C1—N1—C2	127.79 (16)	C2—C7—C6	119.87 (18)
C1—N1—H1N	113.3 (14)	C2—C7—H7	120.1
C2—N1—H1N	117.3 (14)	C6—C7—H7	120.1
C1—N2—N3	117.72 (15)	C5—C8—H8A	109.5
C1—N2—H2N	118.4 (15)	C5—C8—H8B	109.5
N3—N2—H2N	120.2 (15)	H8A—C8—H8B	109.5
C9—N3—N2	117.91 (15)	C5—C8—H8C	109.5
N1—C1—N2	114.54 (16)	H8A—C8—H8C	109.5
N1—C1—S1	125.45 (14)	H8B—C8—H8C	109.5
N2—C1—S1	120.00 (14)	N3—C9—C11	116.21 (16)
C7—C2—C3	119.20 (17)	N3—C9—C10	126.34 (17)
C7—C2—N1	122.98 (17)	C11—C9—C10	117.46 (16)
C3—C2—N1	117.79 (17)	C9—C10—H10A	109.5
C4—C3—C2	120.30 (18)	C9—C10—H10B	109.5
C4—C3—H3	119.9	H10A—C10—H10B	109.5
C2—C3—H3	119.9	C9—C10—H10C	109.5
C3—C4—C5	121.42 (18)	H10A—C10—H10C	109.5
C3—C4—H4	119.3	H10B—C10—H10C	109.5
C5—C4—H4	119.3	C9—C11—H11A	109.5
C4—C5—C6	117.52 (17)	C9—C11—H11B	109.5
C4—C5—C8	121.55 (18)	H11A—C11—H11B	109.5
C6—C5—C8	120.92 (18)	C9—C11—H11C	109.5
C7—C6—C5	121.69 (18)	H11A—C11—H11C	109.5
C7—C6—H6	119.2	H11B—C11—H11C	109.5
C5—C6—H6	119.2		
			
C1—N2—N3—C9	−165.78 (16)	C3—C4—C5—C6	−0.4 (3)
C2—N1—C1—N2	−171.29 (17)	C3—C4—C5—C8	178.82 (19)
C2—N1—C1—S1	9.3 (3)	C4—C5—C6—C7	0.0 (3)
N3—N2—C1—N1	11.0 (2)	C8—C5—C6—C7	−179.16 (18)
N3—N2—C1—S1	−169.57 (12)	C3—C2—C7—C6	−1.1 (3)
C1—N1—C2—C7	−50.1 (3)	N1—C2—C7—C6	−178.81 (17)
C1—N1—C2—C3	132.2 (2)	C5—C6—C7—C2	0.7 (3)
C7—C2—C3—C4	0.8 (3)	N2—N3—C9—C11	−177.62 (15)
N1—C2—C3—C4	178.60 (17)	N2—N3—C9—C10	2.4 (3)
C2—C3—C4—C5	0.0 (3)		

### Hydrogen-bond geometry (Å, º)

Cg1 is the centroid of the C2–C7 ring.

**Table:**

*D*—H···*A*	*D*—H	H···*A*	*D*···*A*	*D*—H···*A*
N1—H1*N*···N3	0.88 (1)	2.09 (2)	2.572 (2)	114 (1)
N1—H1*N*···S1^i^	0.88 (1)	2.87 (2)	3.5618 (17)	137 (2)
N2—H2*N*···S1^ii^	0.88 (2)	2.57 (2)	3.4373 (16)	169 (2)
C8—H8*C*···*Cg*1^iii^	0.98	2.81	3.716 (2)	154

Symmetry codes: (i) *x*, −*y*+1/2, *z*−1/2; (ii) −*x*, −*y*, −*z*+1; (iii) −*x*+1, −*y*, −*z*+1.
